# Effects of First Metatarsophalangeal Osteoarthritis on Plantar Pressures Across Multiple Activities

**DOI:** 10.1002/jfa2.70146

**Published:** 2026-03-19

**Authors:** Scott Telfer, Corey Wukelic, Nicholas Entress, William R. Ledoux

**Affiliations:** ^1^ Center for Limb Loss and MoBility Seattle Washington USA; ^2^ Department of Orthopaedic Surgery and Sports Medicine University of Washington Seattle Washington USA; ^3^ Department of Mechanical Engineering University of Washington Seattle Washington USA

**Keywords:** arthritis, big toe, foot type, pedobarography

## Abstract

**Background:**

The first metatarsophalangeal joint (MTPJ1) is one of the sites most often affected by osteoarthritis (OA). The condition contributes to activity restrictions and reduced quality of life along with biomechanical changes that affect the joint and beyond. Despite this, previous studies have presented limited evidence of alterations in plantar pressures during level walking. In this case‐control study, we hypothesized that activities that may stress the joint to a greater extent than standard gait may reveal greater differences in plantar pressures between those with MTPJ1 OA and healthy feet.

**Methods:**

Participants with MTPJ1 OA (*n* = 22) and matched controls (*n* = 21) were recruited for this study. All were asked to perform four activities while plantar pressures were measured: standing, walking, stair ascent, and heel raise. Analysis of discrete pressure variables at anatomical regions and statistical parametric mapping was used to determine if there were significant differences in pressures between MTPJ1 OA and control groups.

**Results:**

No differences were found for discrete variables across the activities, however, statistical parametric mapping revealed differences for the MTPJ1 OA group, including reduced loading under the distal first metatarsal head (60%–80% of stance), increased midfoot pressures at 70%–80% of stair ascent, and decreased lateral forefoot pressure during heel raise.

**Conclusion:**

MTPJ1 OA is associated with limited changes in plantar pressures across different activities. These regions of difference may be targeted by interventions such as foot orthotics as part of a personalized treatment program.

**Level of Evidence:**

Level IV, case control study.

## 1 Introduction

The first metatarsophalangeal joint (MTPJ1) is an essential component of the kinematic chain for locomotive activities, and impairments to this joint can have significant effects on independence and quality of life [1]. The MTPJ1 is especially prone to degenerative changes in older adults, with osteoarthritis (OA) of the joint impacting around 46% of females and 36% of males over the age of 60, and affecting over 16 million seniors in the United States making this a substantial public health problem [2]. The condition can result in pain, stiffness, swelling, and loss of range of motion at the joint, culminating in restrictions on the individual's ability to perform activities of daily living [1, 3]. Conservative interventions (i.e., physical therapy and orthoses [4, 5]) are the first line of treatment, however a substantial portion of patients ultimately require a surgical procedure to be performed on the joint, with options including cheilectomy [6], arthrodesis [7], or arthroplasty [8].

MTPJ1 OA has been found to be associated with structural changes to the foot, including a dorsiflexed first metatarsal [9]. Furthermore, the condition has been linked to kinematic changes at the joint [10] as well as secondary effects on overall gait patterns [11]. Given that it is estimated that the peak load on MTPJ1 during gait can be equivalent to approximately twice body weight [12], we would anticipate seeing widespread biomechanical changes in foot function with MTPJ1 OA.

The majority of previous studies investigating foot function in those with MTPJ1 OA have looked at walking over level ground. In studies comparing plantar pressures in healthy individuals to those with MTPJ1 OA this has led to mixed results with some studies reporting limited changes [13, 14] and others finding no differences [15, 16] between groups. Modest associations have been found between plantar time integral and prevalence of MTPJ1 pain [17]. Activity monitoring studies reporting on movements in the real world environment have shown that a large percentage of steps involve different types of locomotion including, but not limited to, navigating stairs [18], turning [19], and shuffle steps [20]. Based on results from studies focusing on other joints [21, 22], we suspect that activities such as stair climbing are likely to produce MTPJ1 joint loads and postures that are more extreme than those encountered during tasks based on level ground walking.

Therefore, we suspect that more functionally challenging activities for the joint may result in clearer differences in these pressure distributions between healthy feet and those with MTPJ1 OA. In this study, we looked to assess whether there are differences in plantar pressure distributions between individuals with MTPJ1 OA and matched controls across several selected activities. We hypothesize that there will be significant differences in plantar pressure distributions between participants with and without MTPJ1 OA when performing these activities.

## 2 Methods

Study design: This was a cross‐sectional study of patients with MTPJ1 OA and age‐ and sex‐matched controls. Participants attended a single laboratory visit during which they underwent clinical and biomechanical assessments.

Participants: Participants with a clinical diagnosis of MTPJ1 OA who reported pain at the joint when walking were recruited from the podiatric clinic at VAPSHCS between July 2022 and January 2025. Patient notes were reviewed for terms such as “hallux rigidus”, “hallux MTP osteoarthritis”, “first MTP osteoarthritis”, and “moderate to severe degenerative changes in the hallux MTP” to identify those who may be suitable for inclusion and subsequently underwent telephone screening for eligibility. Given that most of activities have not previously been tested in this population, the sample size was pragmatic and based on sequential recruitment of patients and matched controls, however the sample is greater or similar to those in other studies looking at level walking [14, 15]. Controls were recruited from the general patient population at the VAPSHCS. All participants provided written informed consent upon enrollment. Potential participants were considered ineligible for inclusion if they had any recent (previous 12 months) or current surgical, neurological, metabolic, or musculoskeletal condition considered likely to impair ambulation, inflammatory arthritis with multiple joint involvement, or complicated neuromusculoskeletal treatment procedures requiring multiple corrections not associated with MTPJ1 OA.

Clinical exam: All participants had their feet examined by a qualified clinician. Foot posture was determined by clinical exam, and the range of motion (plantar/dorsiflexion) was measured for both MTPJ1 joints using a goniometer with the foot off the ground in a neutral position [23]. For each participant, one foot was selected for biomechanical testing. In the MTPJ1 OA group, this was the affected foot or, in cases where both feet had diagnosis of MTPJ1 OA, the most affected foot as assessed by joint range of motion; for the control group the foot was randomly chosen.

Equipment: An emed plantar pressure measurement platform (novel GmbH, Munich, Germany) with a resolution of 4 sensors per cm^^2^ and sampling at 100 Hz was embedded level with the floor surface. This system has been validated for capturing plantar pressures [24] and was used to collect data for all of the described activities.

Activities: Barefoot plantar pressure measurements were recorded with the participants performing the following activities:

1. Standing. The participant was asked to step onto the pressure plate and stand in a relaxed standing pose for 6 s. The final 4 s of the trial were used for analysis.
2. Walking. The participant was asked to walk in a straight line over a level surface that included the pressure plate. Participants were instructed to look ahead and not to target the plate. The step was defined as the duration of contact between the foot and the plate. Walking speed was controlled within ± 10% of a self‐selected target.
3. Stair ascent. The stair ascent task consisted of a single step 17 cm in height [25] that the participants were asked to approach at a steady speed from approximately 6 m away. The pressure plate was embedded on the ground in front of the step, therefore the foot of interest was the lower (push‐off) foot. The step was defined as the duration of contact between the foot of interest and the pressure plate.
4. Bilateral heel raise. The participant was asked to step onto the pressure plate, then go up as high as they could on their tip toes as if they were reaching for something high, then return to foot flat before stepping off the plate. The timeframe of interest was defined as beginning when the heel ceased to be in contact with the plate, and ending at the timepoint when the heel re‐contacted the plate. Support was available if necessary, but all participants were able to complete the movement independently.

For all activities, participants were allowed as many practice trials as required to achieve a smooth and natural motion, as judged by an experienced researcher. With the exception of the standing trial, we aimed to capture 5 successful trials for each activity, defined as the movement being performed correctly and in a controlled manner; the foot falling within the bounds of the sensing area on the pressure plate; and the task being performed at the predefined target speed ( ± 10%).

Data processing: Pressure data were processed in the R statistical software language with extensive use of the pressuRe package [26]. Group characteristic data was compared between groups using the *t*‐test or Chi‐squared test as appropriate. Static and dynamic (from walking trials) arch indexes [27] and center of pressure excursion indexes [28] were calculated for all participants as measures of foot type.

Pressure data for all of the activities were analyzed in two ways to allow a comprehensive comparison to be performed. First, discrete pressure variables for anatomical regions of the foot were assessed. All footprints were divided into masked areas corresponding to anatomical regions (hallux, lesser toes, metatarsal heads 1–5, forefoot (all metatarsal head regions combined), midfoot, and heel). Peak pressure, defined as the highest detected value by any single sensor in the mask during the time course of the measurement, and pressure time integral, defined as the quotient of the force time integral over contact area during the measurement [29], were calculated for each mask during each trial. These variables were subsequently compared using independent two‐sided *t*‐tests corrected for multiple comparisons (initial *α* = 0.05, Holm method used to reduce the likelihood of false positives while preserving statistical power) and 95% confidence intervals determined.

Despite the previously described approach for assessing plantar pressure data being commonly reported in the literature [30], limitations have been shown with discrete variables due to sensitivity to boundary conditions and the reductive nature of these analyses [31]. Therefore, as a secondary assessment statistical parametric mapping was used to assess the time series pressure data for all of the activities. The process is described in detail elsewhere [32], but briefly, for walking, stair ascent, and heel raise trials, 3D arrays containing the pressure data were resampled to 101 time points. To allow for comparisons across all feet/groups, a mean footprint was generated for each participant/activity by aligning all five trials to a reference footprint using a translational and rotational registration approach implemented in the RNiftyReg package [33]. Subsequently these mean footprints were all similarly aligned to a single template footprint using the same approach with the addition of scaling into the registration algorithm. We repeated this process for several randomly chosen template feet to ensure the results were not sensitive to the initial template. After registration of all steps, active pixels were compared between groups using statistical parametric mapping to calculate the t‐statistic for each timepoint. To allow reasonable visualization of the results, t‐statistics were summarized for each 10% interval of the activity, and areas where significant differences were detected indicated.

## 3 Results

Twenty‐two individuals with MTPJ1 OA and 21 age and sex matched controls took part in this study. Baseline characteristics and demographic information of both groups were recorded (Table 1). In terms of demographics, the MTPJ1 OA group was found to be shorter (*p* = 0.02) than the control group. For foot mechanics and posture, the MTPJ1 OA group had a smaller MTPJ1 range of motion in the dorsiflexion direction (*p* < 0.001) than the controls, and there was no difference in either the clinical or footprint‐based measures of foot type.

**TABLE 1:** Participant group characteristics.

	MTPJ1 OA	Control	*p*‐value [95% CI]
*n*	22	21	—
Sex	5F/17M	5F/16M	0.95
Age (years)	68.2 (8.0)	62.6 (14.6)	0.14 [−13.1, 1.9]
Mass (kg)	87.0 (14.4)	87.5 (18.8)	0.95 [−10.0, 10.7]
Height (m)	1.70 (0.09)	1.76 (0.09)	0.02 [0.01, 0.12]
Foot side (right/left)	L: 9; R: 13	L: 7; R: 14	0.86
Walking speed (m/s)	1.07 (0.21)	1.16 (0.16)	0.07 [−0.01, 0.23]
MTPJ1 range of motion (plantarflexion)	31.4 (18.5)	32.4 (13.2)	0.85 [−9.3, 11.2]
MTPJ1 range of motion (dorsiflexion)	51.2 (13.9)	68.0 (15.7)	< 0.001 [7.4, 26.2]
Foot type (clinical exam)	Cavus: 2; neutral: 8; planus: 12	Cavus: 1; neutral: 14; planus: 6	0.14
AI (static)	0.25 (0.09)	0.22 (0.07)	0.27 [−0.08, 0.03]
AI (dynamic)	0.27 (0.05)	0.25 (0.05)	0.27 [−0.05, 0.01]
CPEI	20.7 (10.4)	24.9 (11.2)	0.22 [−0.03, 0.11]

Abbreviations: AI: arch index (larger values are associated with planus foot type, smaller with cavus); CPEI: center of pressure excursion index (larger values are associated with “supinated” foot types, smaller with “pronated”); MTPJ1: first metatarsophalangeal joint; OA: osteoarthritis.

**FIGURE 1:**
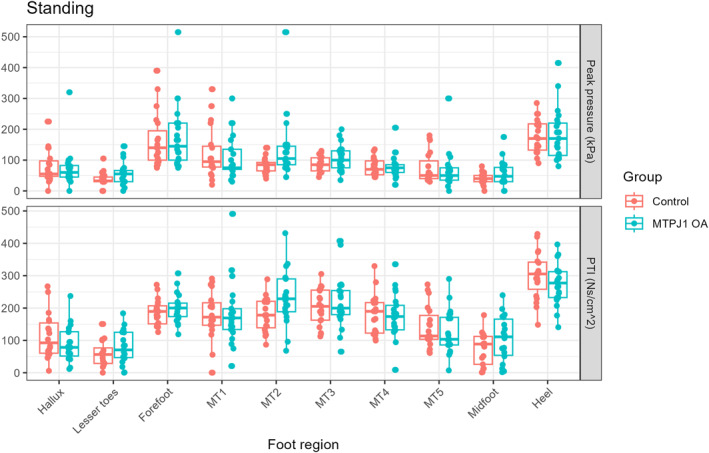
Peak pressure (top) and pressure time integral (PTI; bottom) results for standing activity.

**FIGURE 2:**
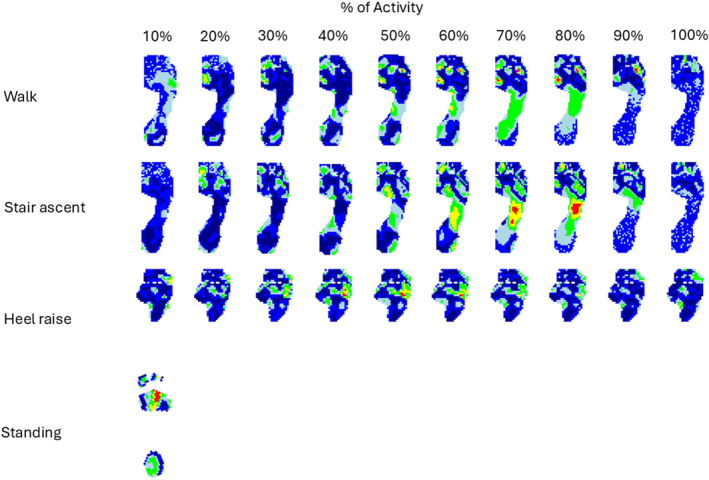
Statistical parametric mapping results for all activities. Left to right represents timeframes of activity divided into 10% (with the exception of standing). Areas indicated in red show statistically significant differences (*p* < 0.05).

Standing: For the static pedobarographic measurement, no differences were found between the MTPJ1 OA and control groups when assessing the discrete masked variables (Figure 1). Statistical parametric mapping suggested differences at the second and third metatarsal heads (Figure 2).

**FIGURE 3:**
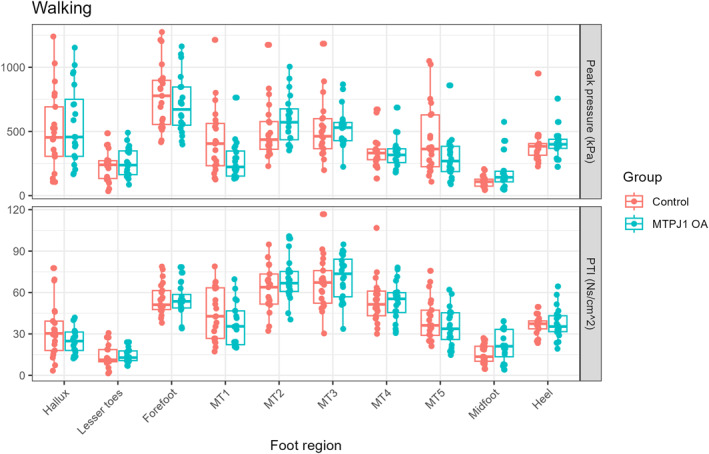
Peak pressure (top) and pressure time integral (PTI; bottom) results for level walking activity.

Walking: Discrete pressure variables were not found to be significantly different between groups during level walking (Figure 3). Using statistical parametric mapping, we saw differences at metatarsal head 1 around the 60%–80% phase of stance, as well as the lesser toes around similar time points (Figure 2).

**FIGURE 4:**
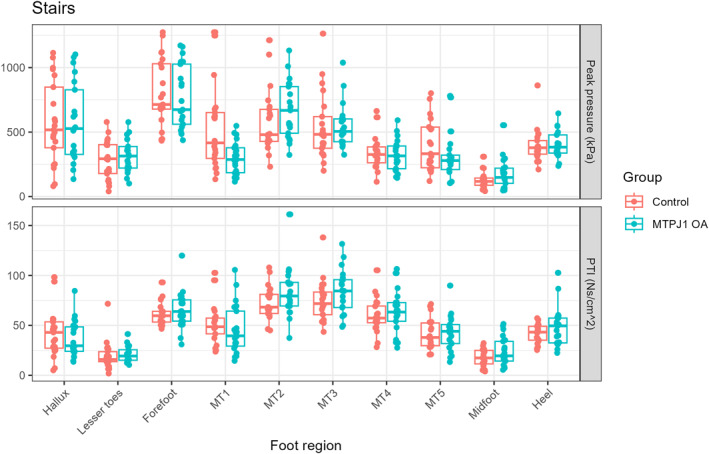
Peak pressure (top) and pressure time integral (PTI; bottom) results for stair ascent activity.

Stair climb: Similarly, no differences were found in the discrete pressure variables for this activity (Figure 4). Statistical parametric mapping found that there were significant differences between groups at the midfoot during the 70%–80% of the step (Figure 2).

**FIGURE 5:**
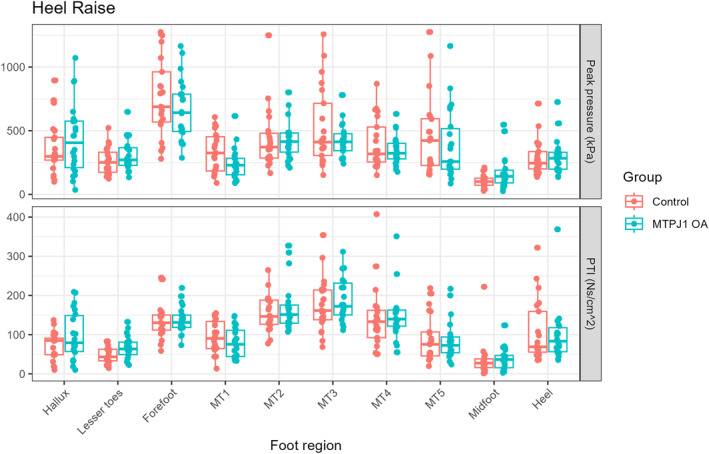
Peak pressure (top) and pressure time integral (PTI; bottom) results for heel raise activity.

Heel raise: No significant differences were detected between groups for the discrete pressure analysis (Figure 5). Statistical parametric mapping however suggested differences at the distal part of the lateral forefoot around 40%–60% of the cycle (Figure 2).

## 4 Discussion

In this study, we report on differences in plantar pressures between patients with MTPJ1 OA and healthy controls across several activities. We found that MTPJ1 OA had some limited effects on plantar pressures. Our original hypothesis was that activities which were likely to be more challenging for MTPJ1 OA patients (such as stair ascent and heel raises) would result in greater differences in plantar pressures than have been observed previously. Although we did see some differences, these were relatively small and it may be challenging to draw conclusions from at the group level.

In our study, we found no differences using “traditional” pressure distribution analysis techniques involving discrete variables, however statistical parametric mapping, which has gained in popularity in recent years [32, 34], did reveal subtle differences in between the groups. Although discrete variables have been found to have some limitations [31], these are still commonly reported and do allow for some intuitive anatomical understanding of the differences in loading patterns [35].

In line with our results, Stevens et al. reported no significant changes in pressure time integral between MTPJ1 OA and control subjects despite other kinematic changes in gait being noted [15], and Miana reported similar findings [16]. Studies comparing plantar pressures in patients with MTPJ1 OA pre‐ and post‐surgery have reported increases at the first metatarsal head during gait after the intervention, similar to what we found using statistical parametric mapping [7, 36]. This offloading of the first metatarsal head in MTPJ1 OA may be a result of pain at this site and require compensatory changes at other sites in the foot. Zammit et al. found that peak pressures were 23% higher beneath the hallux and 29% greater beneath the lesser toes, but no other regions were affected in those with radiographically confirmed MTPJ1 OA [14]. However, we note that the reported peak pressures are 3–4 times lower than we found, suggesting that a different definition of peak pressure was used. This ambiguity in the reporting of plantar pressure variables has been flagged elsewhere [26]. The changes seen at the midfoot during stair ascent may reflect an unwillingness to extend the first metatarsophalangeal joint during this activity and place greater stress on other parts of the kinetic chain. This is in line with findings from Rao et al. who suggested MTPJ1 OA patients may use gait strategies to avoid pain [17].

In the present study, we did not find any significant differences between MTPJ1 OA and controls for foot type using any clinical or footprint‐based measures, although the MTPJ1 OA feet showed a slight trend toward being more planus. This is in line with a previous systematic review of the literature where Zammit et al. did not find any differences to be reported between foot posture and arch height in MTPJ1 OA patients and controls [9], although Menz et al. did report on differences in center of pressure patterns [13].

MTPJ1 OA remains a pressing musculoskeletal health issue. Radiographically confirmed MTPJ1 OA was found to affect 25% of patients having emergency room visits for foot injuries [37]. Early clinical interventions for MTPJ1 OA are largely conservative, with non‐steroidal anti‐inflammatory drugs, foot orthoses, and intra‐articular steroid injections all showing some benefit [38]. Orthotic and footwear interventions have received increased attention in recent years will some promising findings [39, 40, 41]. The results of the present study may help to inform the design of orthotic and footwear‐based interventions, as the ability to use plantar pressure data to optimize these devices has been demonstrated in other patient populations [42, 43]. We note significant variation across the results in the MTPJ1 OA group, which may suggest the need to identify those with elevated pressures who may benefit from these types of interventions.

There are limitations to be noted in this study. These assessments were performed with the subjects barefoot. It is likely that the use of different types of shoes would influence the effects we saw and may alter the results. The diagnosis of MTPJ1 OA in the study population was clinically rather than radiologically confirmed, however the majority of the participants matched the dorsiflexion range of motion threshold used in previous studies [14]. Walking speed has been found to affect plantar pressure measurements [30], and in this study the control group trended toward walking faster than the patient group. However, we would expect any substantial effects from MTPJ1 OA to still be reflected in the pressure time integral variables. Disease specific aspects such as duration and pain levels were not captured as this study involved a pragmatic sample of MTPJ1 OA patients, and functional restrictions were our primary measure of disease impact. Finally, a larger sample size may reveal clearer differences between the groups.

## 5 Conclusion

In this study, individuals with a diagnosis of MTPJ1 OA were found to have subtle changes in their plantar pressures across multiple activities. It has been suggested that these types of changes in plantar pressure loading may lead to secondary complications such as callus formation and additional strain on the damaged joint [14]. Given the known mechanical changes at the joint, we suspect that individuals with MTPJ1 OA may have secondary changes in gait that are used to compensate for changes in the structure and function of MTPJ1 and avoid overloading the regions around the joint.

## Author Contributions

**Scott Telfer:** conceptualization, data curation, formal analysis, funding acquisition, investigation, methodology, software, visualization, writing – original draft preparation, writing – review and editing. **Corey Wukelic:** data curation, investigation, writing – review and editing. **Nicholas Entress:** data curation, investigation, writing – review and editing. **William R. Ledoux:** conceptualization, data curation, funding acquisition, investigation, methodology, project administration, supervision, writing – review and editing.

## Funding

This research was supported by US Department of Veterans Affairs, Rehabilitation Research and Development Service Grant Nos. RX003259 and RX002970.

## Ethics Statement

The VA Puget Sound Health Care System (VAPSHCS) Institutional Review Board approved all procedures prior to the study commencing and participants provided informed consent.

## Conflicts of Interest

The authors declare no conflicts of interest.

## Data Availability

The data that support the findings of this study are available from the corresponding author upon reasonable request.
